# MircoRNA-26a与肿瘤

**DOI:** 10.3779/j.issn.1009-3419.2017.11.08

**Published:** 2017-11-20

**Authors:** 倩倩 宋, 克 徐

**Affiliations:** 300052 天津，天津医科大学总医院，天津市肺癌研究所，天津市肺癌转移和肿瘤微环境重点实验室 Tianjin Key Laboratory of Lung Cancer Metastasis and Tumor Microenvironment, Tianjin Lung Cancer Institute, Tianjin Medical University General Hospital, Tianjin 300052, China

**Keywords:** miR-26a, 靶基因, 肿瘤, miR-26a, Target genes, Tumors

## Abstract

微小RNA（microRNAs, miRNAs）是一类由20个-22个核苷酸组成的小片段非编码RNA，通过靶向结合基因mRNA的3’非翻译区（3’-UTR）调控其表达。许多研究报道miRNAs参与肿瘤的发生发展。MiR-26a在不同的肿瘤中发挥不同的作用，在肿瘤增殖、转移侵袭、血管形成、生物代谢及诊断预后中都有作用。本文就miR-26a与肿瘤关系的研究进展进行综述。

## Abstract (en)

MicroRNAs are a class of small noncoding RNAs (20-22 nucleotides). MicroRNAs regulate gene expression by directly binding gene's 3'-UTR regions. Many studies reveal that miRNAs play roles in tumorigenesis. MiR-26a plays different roles in different types of cancer, and participates in tumor growth, invasion, metastasis, angiogenesis, metabolism, diagnosis and prognosis. This review summarizes the progress of miR-26a study in cancer.

肿瘤作为目前世界上导致死亡的主要疾病之一，对人类健康造成了极大威胁，而且由于其治疗手段和治疗效率的局限性，目前肿瘤治疗的目的之一是延长患者寿命和提高患者生存质量^[1]^。肿瘤是由遗传因素（原癌基因激活、肿瘤抑制基因失活和端粒酶活性存在等）和其他外界因素（射线、化学物质和病毒等）刺激而诱发机体局部组织细胞增生所形成的新生物。肿瘤的发生包括5个阶段：癌前阶段、原位癌、浸润癌、局部或区域性淋巴结转移、转出播散。目前肿瘤的主要治疗手段是手术、放疗和化疗，以及少数的靶向治疗，如：非小细胞肺癌患者服用的靶向药物：表皮生长因子受体（epidermal growth factor receptor, EGFR）酪氨酸激酶抑制剂吉非替尼（gefitinib）和厄洛替尼（erlotinib）。

微小RNA（mircoRNAs）是由20个-22个核苷酸（nt）组成的在细胞内广泛存在的内源性非蛋白编码小分子RNA，通过靶向结合mRNA的3’非翻译区（信使RNA分子3’的非编码片段，3-untranslated region，3’-UTR），导致靶基因表达下降或降解，进而参与许多生命活动的调控^[2]^。通常来说，一种miRNA可以靶向结合调控多个mRNAs，一个mRNA也可能由多种miRNAs调控的。MiRNAs是由含有环状结构的miRNAs的初级转录产物（pri-miRNAs）在细胞核内由Drosha加工成约60 nt-90 nt的miRNAs前体（pre-miRNAs），然后pre-miRNAs通过核输出蛋白5（nuclear output protein 5）途径转运至细胞质，在细胞质中由RNAseⅢ-Dicer剪切为成熟miRNAs，详见图 1。目前已发现的miRNA有28, 600多种，其中人源性miRNA有4, 500多种，许多研究显示很多miRNA在机体生命活动过程中发挥重要作用。成熟的miRNA-26a包括miR-26a-1和miR-26a-2，其中，miR-26a-1位于人染色体3q21.3上，miR-26a-2位于人染色体12q14.1上，miR-26a的成熟体序列为: UUCAAGUAAUCCAGGAUAGGCU。目前研究显示miR-26a在骨损伤修复、成骨分化、糖尿病伤口愈合、脂质代谢及肿瘤的发生发展等过程中均可以发挥重要作用，本文主要就miR-26a与肿瘤生长、转移、耐药、预后及生物治疗的研究进展进行综述。

**1:**
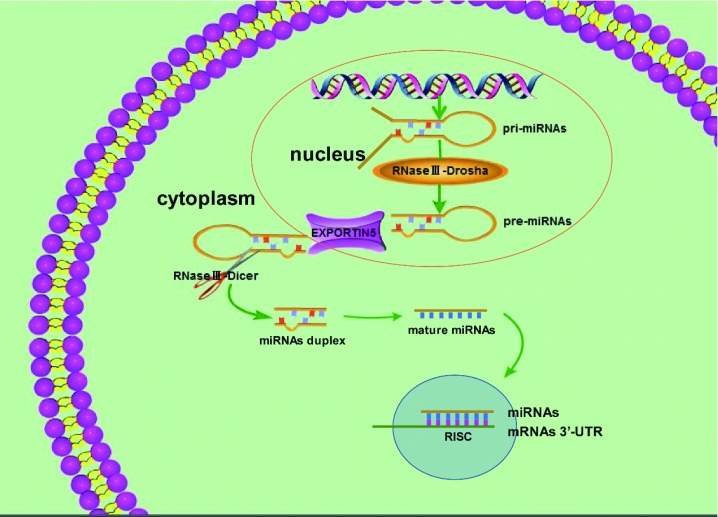
miRNA的合成 The biogenesis of miRNAs

## 1 MiR-26a对肿瘤生长的影响

癌症是由于机体正常细胞失去正常调控，过度增殖而引起的疾病，它的形成是一个复杂的过程。正是由于癌细胞的无限增殖性使得癌症治疗存在很多困难，因此抑制肿瘤细胞增殖对于抑制肿瘤生长起关键作用。有研究显示，miR-26a参与相关基因的转录调节，如：组蛋白甲基转移酶（enhancer of zeste homolog 2, EZH2）、同源性磷酸酶-张力蛋白（phosphatase and tensin homolog, PTEN）、高迁移率族蛋白（high mobility group protein, HMGA）等，因此miR-26a的异常表达可以对细胞生长、G_1_期/S期转化和细胞凋亡产生影响。

### 1.1 MiR-26a对肿瘤细胞生长的影响

在肝癌中，Chen等^[3]^报道miR-26a在甲胎蛋白和人端粒酶逆转录酶的双重调节下高表达，通过靶向抑制雌激素受体*α*（estrogen receptor-*α*, ER-*α*）的表达，进而降低细胞的生存能力。ER-*α*的3’-UTR区可以与miR-26a的种子区有效结合，使得ER-*α*在转录和翻译水平均低表达，抑制肝癌细胞生长。白介素6/信号转导子和转录激活子3（interleukin 6/signal transducer and activator of transcription 3, IL-6/STAT3）是miR-26a在肝癌中作用的另一重要通路，Yang等^[4]^研究显示miR-26a靶向作用于IL-6，通过抑制IL-6/STAT3通路抑制肝肿瘤生长。Zhang等^[5]^报道显示，在肝癌中miR-26a的低表达可能是IL-6表达增高活化c-Myc而导致的。

在肝癌、肺癌、乳腺癌等中发现miR-26a作为抑癌基因存在，相反，在胶质瘤、胆管癌中miR-26a表达水平增高，作为癌基因存在。在急性髓性白血病细胞中miR-26a靶向作用于E2F7维持细胞增殖，抑制单核细胞分化^[6]^。在卵巢癌中，miR-26a靶向抑制ER-*α*的表达促进细胞生长^[7]^。在胆管癌中，w-3不饱和脂肪酸抑制miR-26a表达，靶向诱导15-羟基前列腺素（15-hydroxyprostaglandin dehydrogenase, 15-PGDH）表达从而抑制细胞生长^[8]^。MiR-26a也可以通过靶向作用于糖原合成酶激酶-3β（glycogen synthase kinase-3β, GSK-3β），活化β-连环蛋白（β-catenin）促进胆管癌细胞生长和克隆形成^[9]^。在胶质瘤中，miR-26a靶向作用于抗增殖蛋白（prohibitin）^[10]^和PTEN^[11]^促进胶质瘤细胞生长。

### 1.2 MiR-26a对肿瘤细胞凋亡的影响

EZH2是多梳抑制复合物家族成员，可以使其他基因的组蛋白发生甲基化进而抑制基因翻译。EZH2是miR-26a的直接作用靶点，miR-26a调节其表达出现在许多肿瘤中。Dang等^[12]^发现miR-26a靶向抑制EZH2表达，诱导DOC2/DAB2结合蛋白（DAB2IP）和人类相关转录因子3（RUNX3）表达，抑制肺癌细胞生长，导致G_1_期/S期转化受阻，诱导凋亡。Zhang等^[13]^研究显示miR-26a可以靶向异粘蛋白（metadherin, MTDH）和EZH2抑制乳腺癌细胞增殖，诱导凋亡，同样的作用发生在肝癌^[14]^和鼻咽癌^[15]^中。

Tong等^[16]^研究发现KH型剪接调节蛋白（KH-type splicing regulatory protein, KHSRP）使miR-26a在肺癌中低表达从而使PTEN表达升高，抑制肺癌细胞生长。Ichikawa等^[17]^发现在乳腺癌中miR-26a可以抑制细胞生长，miR-26a的升高与赫赛汀治疗有剂量依赖关系，导致乳腺癌细胞G_1_期停滞，诱导凋亡。在乳头状甲状腺癌中，miR-26a靶向作用于细胞周期蛋白依赖性激酶亚基蛋白2（cyclin dependent kinase subunit 2, GKS2）促进细胞凋亡^[18]^。在急性髓性白血病中，miR-26a表达水平低，外源性高表达miR-26a可以靶向抑制过氧化物还原酶Ⅲ（peroxiredoxin Ⅲ），使活性氧（reactive oxygen species, ROS）水平升高，促进凋亡^[19]^。在胶质瘤中，miR-26a靶向作用于共济失调症突变蛋白（ataxia-telangiectasia mutated, ATM），抑制胶质瘤细胞G_1_期/S期转化^[20]^。二甲双胍通过上调miR-26a的表达，使MCL-1低表达，促进口腔癌细胞凋亡^[21]^。

## 2 MiR-26a在肿瘤侵袭转移中的作用

### 2.1 MiR-26a对上皮间质转化的影响

MiR-26a在淋巴结转移肿瘤中的表达与原发肿瘤有差异，且表达水平与不同的临床分期有关。Ma^[14]^、Wang等^[22]^证实miR-26a可以靶向抑制EZH2表达，通过抑制上皮间质转化（epithelial-mesenchymal transition, EMT）进程，进而抑制肝癌细胞生长和侵袭转移。Yang等^[4]^证实miR-26a可以靶向作用IL-6抑制其翻译，沉默STAT3转录因子，调控一系列基因表达，如：基质金属蛋白酶-2（matrix metalloproteinase-2, MMP-2），髓细胞白血病-1基因（myeloid cell leukemia 1, *MCL-1*），B淋巴细胞瘤-2基因（B-cell lymphoma 2, *BCL-2*）和G_1_/S-特异性周期蛋白-D1（Cyclin D1）等，抑制肝癌细胞在体内及体外的生长和转移。Liu等^[23]^发现在肺癌转移淋巴结中miR-26a表达水平比原发肿瘤组织高，miR-26a可以靶向抑制PTEN表达，通过蛋白激酶B（protein kinase B, Akt）通路促进肺癌细胞转移。成纤维细胞趋化因子（fibroblast chemotactic factor, *FGF*）是作为癌基因促进EMT发生的，能在很多恶性肿瘤中刺激肿瘤侵袭转移。在胃癌中，miR-26a靶向抑制FGF9表达，抑制胃癌细胞生长和转移^[24]^。在乳腺癌中，miR-26a抑制MCL-1表达，抑制细胞生长和转移，增加细胞对紫杉醇的敏感性^[25]^。

### 2.2 MiR-26a对血管生成的影响

肿瘤作为一种消耗性疾病，肿瘤细胞的无限生长特性要求肿瘤环境血供丰富，为其生长提供充足的营养物质，因此血管生成对于肿瘤的生长和转移十分重要。包括miR-26a在内的miRNAs可以调节促血管生成和抗血管生成的因子从而影响肿瘤的血管生成。

血管内皮生长因子A（vascular endothelial growth factor A, VEGFA）可以促进血管生成，促进肿瘤侵袭和转移。在肝癌中，miR-26a靶向调控VEGFA的表达，通过抑制PIK3C2*α*/Akt/HIF-1*α*通路，进而抑制肿瘤血管生成^[26]^。肝细胞生长因子（hepatocyte growth factor, HGF）是miR-26a在肝癌中的另一作用靶点，miR-26a通过抑制HGF-cMet通路抑制肿瘤血管生成，miR-26a调节HGF-cMet的作用可以抑制PI3K/Akt/mTOR/S6K和HIF-1*α*-VEGF信号通路，抑制肝癌的血管生成^[27]^。

### 2.3 MiR-26a对转移相关基因的调控

HMGA是一种带有3个AT-hook结构域的核基质蛋白，参与调控细胞周期转换及细胞活动性，是肿瘤细胞转移的动力。MiR-26a在乳腺癌组织中低表达，外源性高表达miR-26a可直接靶向抑制HMGA1，进而抑制乳腺癌细胞生长，抑制肿瘤侵袭转移^[28]^。MiR-26a在膀胱癌中低表达，可靶向抑制HMGA2，抑制膀胱癌细胞增殖，且miR-26a的低表达与膀胱癌的组织学分期相关，miR-26a的低表达与差的病理分期有关^[29]^。在肺癌中，miR-26a与HMGA1表达呈负相关，miR-26a可靶向抑制HMGA1的表达，抑制肺癌细胞的侵袭转移^[30]^。

### 2.4 MiR-26a对失巢凋亡的影响

失巢凋亡是一种当细胞与其周围基质和邻近细胞分离后而被启动死亡的过程，因此肿瘤细胞逃脱失巢凋亡是其转移的一个重要进程。MiR-26a抑制整合素*α*5（integrin *α*5, ITGA5）在失巢凋亡过程中起重要作用，可能是转移肝癌的治疗靶点^[31]^。在食管癌中，miR-26a靶向Rb1-E2F1通路参与获得性的失巢凋亡抵抗，参与肿瘤形成和转移^[32]^。MiR-26a靶向赖氨酰氧化酶相关蛋白2（lysine oxidase associated protein 2, LOXL2）抑制头颈部鳞癌的生长转移^[33]^。

## 3 MiR-26a在肿瘤代谢中的作用

肿瘤细胞与正常细胞相比有着不同的能量代谢表型，多通过糖酵解途径消耗更多的葡萄糖，产生大量乳酸，而不是通过氧化磷酸化途径。肿瘤细胞的这种对糖酵解通路产能增强的现象被称为“有氧糖酵解”或“瓦博格效应”^[34]^。肿瘤作为消耗性疾病，除了充分的血供提供氧气外，还需要大量的能量以维持其生长，因此，抑制肿瘤产能，抑制其新陈代谢可以有效抑制肿瘤的生长。近来，许多研究表明miRNAs与肿瘤能量代谢存在关系，在有氧糖酵解中起重要作用。MiR-26a也与肿瘤能量代谢有关。在结肠癌中，miR-26a表达升高，可以靶向抑制丙酮酸脱氢酶复合物（pyruvate dehydrogenase complex component X, PDHX），抑制丙酮酸向乙酰辅酶A的转化，促进糖消耗，促进有氧糖酵解以满足结肠癌生物合成和能量代谢^[35]^。

## 4 MiR-26a在临床中的应用

### 4.1 MiR-26a在肿瘤诊断和预后中的应用

肿瘤的发生由多种因素引起，它的发展及预后直接关系到患者的治疗效果，因此肿瘤的早期诊断和早期治疗对于患者的预后生存十分关键。肿瘤标志物现已应用于很多肿瘤的筛查，但是肿瘤标志物多因它的敏感性而使结果不准确。MiRNAs在不同肿瘤组织中的异常表达也成为人们研究的重点，可以与肿瘤标志物结合检测，有利于肿瘤的筛查和预后判断。

相比正常肝组织来说，miR-26a在肝癌中低表达，抑制肝癌细胞生长和巨噬细胞富集，miR-26a低表达与肝癌预后差相关^[36]^。Yang等^[4]^运用实时荧光定量聚合酶链反应（qRT-PCR）分别检测了肝脏正常组织标本和肿瘤组织标本、未转移的肿瘤标本和转移标本、初次发生肿瘤和复发肿瘤的miR-26a表达水平，发现miR-26a表达量在非肿瘤组织表达高于肿瘤组织、未转移组织miR-26a表达量高于转移组织、初次发生肿瘤miR-26a表达量高于复发肿瘤。在淋巴细胞性白血病中，外源性miR-26a高表达靶向抑制PTEN表达，与binet分期、p53异常、首次治疗效果差相关^[37]^。在直肠癌患者中，血浆miR-26a表达与正常人相比下降，与miR-142-3p的表达水平相结合可以作为肠癌的诊断指标^[38]^。MiR-26a在低分化的胶质瘤中表达比高分化的胶质瘤高，提示miR-26a的表达水平可以作为胶质瘤预后判断的指标^[11]^。在骨肉瘤中，外源性高表达miR-26a可以减少骨肉瘤细胞干细胞样特性，抑制肿瘤形成和化学耐药，miR-26a的低表达与骨肉瘤的预后差相关，可以作为骨肉瘤患者的预后标志^[39, 40]^。低表达miR-26a与胃肿瘤转移和复发有关^[41]^。MiR-26a在胃癌组织和血浆中与正常组织和正常人血浆相比，表达量较低，与患者的预后良好及5年生存率有关^[24, 42, 43]^。MiR-26a在分化差的膀胱癌中表达量相比分化好的低，miR-26a的低表达水平与肿瘤预后差相关^[29, 44]^。在胆管癌患者中，血清miR-26a的表达比正常对照高，术后miR-26a水平比术前低，miR-26a高水平表达与临床分期，远处转移，病理分型，预后差有关^[45]^。在胶质瘤中，miR-26a的表达与细胞对射线的敏感性呈正相关，与DNA修复有关^[20]^。

### 4.2 MiR-26a在肿瘤耐药中的作用

肿瘤的耐药作用是每个肿瘤患者治疗过程中的必经环节，肿瘤耐药性的出现给肿瘤的治疗带来了极大的困难。MiR-26a在耐酪氨酸激酶抑制剂的肺癌细胞中的表达比不耐药的细胞高，外源性高表达miR-26a可以靶向抑制酪氨酸蛋白磷酸酶非受体型13（tyrosine-protein phosphatase non-receptor type 13, PTPN13）的表达，增加肺癌细胞对酪氨酸激酶抑制剂的耐药性^[46]^。MiR-26a在顺铂耐药的A549/DDP细胞系中表达比正常A549低，外源性高表达miR-26a可以靶向抑制HMGA2，通过miR-26a-HMGA2-E2F1-Akt-Bcl通路增加细胞对CDDP的敏感性^[47]^。在乳腺癌组织中，miR-26a表达比正常乳腺组织低，外源性高表达miR-26a靶向抑制MCL-1，增加乳腺癌细胞对紫杉醇的敏感性^[25]^；在乳腺癌治疗过程中，miR-26a的表达增高与赫赛汀有剂量依赖关系^[17]^；外源性高表达miR-26a可以靶向抑制EZH2，抑制CDC2表达，利于它莫西芬对转移乳腺癌的治疗^[48]^。在肝癌中，miR-26a表达降低，外源性高表达miR-26a抑制细胞生长和转移。干扰素治疗后miR-26a升高，提示miR-26a可以作为干扰素治疗效果的评价指标^[22]^。在胃癌中，miR-26a靶向抑制神经母细胞瘤病毒致癌基因*RAS*（neuroblastoma RAS viral oncogene homolog, *NRAS*）和*E2F2*，增加胃癌细胞对顺铂的敏感性^[49]^。

## 5 结语

肿瘤是目前世界上发病率和死亡率均位列第一的疾病，许多研究致力于揭示肿瘤发生的病因及发现有效的治疗手段，然而，肿瘤发生、转移及耐药机制仍然未研究清楚。研究发现很多肿瘤组织和肿瘤细胞中，miR-26a的表达水平与正常组织相比存在显著差异。MiR-26a在不同的肿瘤组织中发挥着不同的作用，且在肿瘤发生发展的不同阶段，包括：增殖、转移、血管形成、生物代谢、耐药性及诊断预后中起不同的作用。有研究显示，肿瘤患者血清miR-26a可以检测出差异，术前术后及药物治疗前后miR-26a的表达水平有显著差异，提示miR-26a未来可以作为肿瘤标志物及患者治疗有效性的检测。同时miR-26a在不同分期患者的表达水平的差异提示miR-26a对患者预后判断有意义。

总之，随着对miR-26a的研究不断深入，miR-26a在肿瘤发生发展中的作用机制会逐步揭示。MiR-26a不仅可以作为肿瘤诊断预后的指标，还可以成为肿瘤治疗的有效靶点，为肿瘤治疗提供新方向。
